# Comparative Analysis of Elderly Patients Undergoing Radical Cystectomy With Ureterocutaneostomy or Ileal Conduit With a Special Focus on Bowl Complications Requiring Surgical Revision

**DOI:** 10.3389/fsurg.2022.803926

**Published:** 2022-06-10

**Authors:** David Mally, Patricia John, David Pfister, Axel Heidenreich, Peter Albers, Günter Niegisch

**Affiliations:** ^1^Department of Urology, University Hospital, Medical Faculty, Heinrich-Heine University Düsseldorf, Düsseldorf, Germany; ^2^Department of Urology, University Hospital of Cologne, Cologne, Germany

**Keywords:** radical cystectomy and urinary diversion, radical cystectomy and complications, aging population, elderly people, choice of operating mode, bladder cancer, robotic surgery

## Abstract

**Objectives:**

Ileal conduits (ICs) carry an additional perioperative complication risk due to the bowel procedure. This analysis compares surgical outcomes in patients ≥75 years of age with ureterocutaneostomy (UCN) and IC after cystectomy (Cx).

**Methods:**

Data of 527 patients included in a retrospective cystectomy database of two high volume centers (2008–2020) were queried to identify elderly patients (≥75 years) who underwent Cx either with IC or UCN. Patient characteristics of all patients [age, BMI, Charlson Comorbidity Index (CCI)], perioperative parameters (operation time, blood loss, transfusions, tumor stage), and postoperative complications (clavien >IIIA, intensive care unit (ICU) stay) were compared. As special focus, bowel complications requiring surgical revision (rBCs) were analyzed. In patients with IC, the rate of ureteral implantation stenosis (USt) was recorded. As a population of special interest, patients ≥80 years of age were analyzed separately. Categorical data were compared using Fisher exact test, and continuous data were compared using Mann–Whitney U test.

**Results:**

A total of 163 patients ≥75 years of age (125 IC, 38 UCN) were identified. Patients with UCN were older and presented with a higher CCI, though differences were not statistically different. Surgery with palliative intent was more frequent in patients with UCN (37 vs. 10%). Operation time in UCN was significantly shorter (233 vs. 305 min, *p* = 0.02), while blood loss and transfusion rate were comparable. Overall complication rate (Clavien-Dindo grade IIIA–IVB) was comparable (UCN 34% vs. IC 37%). However, rBC was a rare complication in UCN (3/38) as compared to patients with IC (15/125). Frequency of postoperative ICU stay (UCN 16% vs. IC 16%) and 90-day mortality did not differ (UCN 3/38 patients, IC 5/125 patients). Regarding long-term follow-up, USt requiring revision or permanent stenting was seen in 18/125 (14%) patients with IC. In patients >80 years of age, results were comparable to the main cohort. Low event rate regarding complications and bias inherent of a retrospective analysis (selection bias, unequal distribution in case numbers) precludes detection of statistical differences regarding patients' characteristics and overall complication rate.

**Conclusion:**

UCN is an alternative to IC in elderly and/or frail patients. Severe bowel complications are numerically less frequent and operation time is minimized.

## Introduction

Urothelial bladder cancer is (UBC) is one of the most common cancers worldwide, accounting for approximately 550,000 new cases annually (1). Patients who present with a muscle invasive tumor or with a high-risk non-muscle invasive tumor which can't be controlled by conservative standard treatment, radical cystectomy is the standard curative treatment. As a urinary diversion, continent (e.g., neobladders or pouches) and incontinent [e.g., ileal conduit (IC) or ureterocutaneostomy (UCN)] procedures can be distinguished. The decision of urinary diversion is made between the surgeon and the patient, while it must be considered that not every patient is suitable for a continent urinary diversion regarding haptic abilities or nursing capabilities. Furthermore, several contraindications, for example, impaired renal function, urinary stress incontinence or damaged rhabdomyosphincter or incompetent urethra may impact choice of diversion (2). Incontinent urinary diversion is easier to handle and therefore the better solution for the large part of the patients especially because reoperation rates are lower as compared to continent diversions.

As UBC is a disease of the elderly with a rising incidence with age, the majority of the patients are ≥75 years of age (3). Regarding this fact, physicians have to face not only increasing perioperative morbidity but also decreasing dexterity and a higher chance of contraindications. Incontinent urinary diversion therefore is the standard diversion for the elderly (4). The most common incontinent diversion is the IC (4). The radical cystectomy holds a 90-day complication rate from 50 to 64% (5, 6). As the IC requires a bowel procedure, it carries an additional surgical risk. This risk includes complications which can be maintained conservatively such as prolonged ileus or wound dehiscence (7), but may also result in more life-threatening complications with unplanned surgery such as insufficiencies of the ileal anastomosis or mechanical ileus (8, 9). It is unclear, whether this additive bowel procedure leads to increasing perioperative complications when performing an IC compared to UCN.

The choice of urinary diversion differs on multiple levels. In addition to the abovementioned surgical risks, the expertise of the surgeons or the ability of the patients to endure long anesthesia, and the associated necessity of a short operating time, the long-term complications of the urinary diversion also play a decisive role here. In addition to the abovementioned complications of the IC, these complications include bacterial colonization, stoma-related complications such as prolapse, retraction, or stone formation (7). UCN also tends to bacterial colonization and stenosis of the stoma whereas for the prevention of stomal stenosis, a stent implantation can be performed (10).

In this context, we searched to evaluate perioperative complication rates in elderly patients undergoing radical cystectomy either with IC or UCN. A special focus of this retrospective analysis was gastrointestinal complications requiring further surgery.

## Materials and Methods

### Design, Setting, and Participants

A total of 527 patients were included from 2008 to 2020 in retrospective cystectomy databases of two high volume centers, Dusseldorf and Cologne (Dusseldorf 2008–2020, Cologne 2016–2018). These databases were queried to identify elderly patients [above 75 years of age (11)] who underwent radical cystectomy either with IC or UCN. From these databases, 163 patients met the above requirements, 125 patients received IC, 38 received a UCN. Operation techniques and the presence of a urothelial carcinoma were not a selection criteria. Cystectomy was performed either open or robot–assisted, following a standardized operative treatment (12). Urinary diversion was performed open in every patient. The type of anastomosis was dependent on patients' factors (length of ureter post-cystectomy, ureter tissue status, presurgical ureter dilation) and left to the surgeon's discretion. The cutoff of 75 years was chosen in line with the current literature (13–16). Choice of urinary diversion was based on regular case-based preoperative conferences and individual discussion with the patient and the surgeon. Patients with UCN were usually permanently treated with mono-J catheters, requiring catheter exchanges every 4–6 weeks.

From those 163 patients, detailed data were extracted (see Table 1). Patients' characteristics [age, BMI, Charlson Comorbidity Index (CCI)] and perioperative parameters (operation time, blood loss, transfusion rate, tumor stage, length of hospital stay) as well as postoperative complications [Clavien–Dindo classification, postoperative intensive care unit (ICU) stay] were compared. Additionally, in the IC group, the rate of ureteral stenosis requiring either permanent stenting or revision was recorded.

**Table 1:** Baseline characteristics overall population.

**Urinary diversion type**	**UCN**	**IC**	***p* value**
**Baseline**			
*n*	38	125	
Age, year, median (range)	81 (75–89)	79 (75–89)	0.12
Body mass index, kg/m^2^, median (range)	26 (19–33)	26 (19–37)	0.51
Charlson-Comorbidity-Index, median (range)	7 (5–16)	6 (0–11)	0.75
Palliative intention, *n* (%)	14 (37)	13 (10)	0.04
**Perioperative**			
Operation time, min, median (range)	236,5 (130–450)	277,5 (100–600)	0.02
Transfusion rate, *n* (%)	12 (32)	33 (26)	0.69
Blood loss, ml, median (range)	300 (0–1200)	300 (0–6000)	0.77
Hospital stay, d, median (IQR)	18 (14,75)	17 (10)	0.96
Intensive care unit stay, *n* (%)	9 (24)	19 (15)	1
Intensive care unit stay, d, median (range)	5,5 (1–9)	3 (1–24)	0.86
Pelvic lymph node dissection, *n* (%)	16 (42)	99 (79)	0.14
Lymph node yield, *n*, median (range)	8 (2–36)	17 (1–47)	0.006
**Postoperative**			
Tumor stage, *n* (%)			
T0, ≤ T1	9 (24)	34 (27)	0.67
T2	7 (18)	27 (22)	0.67
T3	12 (32)	34 (27)	0.59
T4	10 (26)	30 (24)	0.77
Neoadjuvant chemotherapy, *n* (%)	0 (0)	7 (6)	0.53
Follow-up, m, median, IQR (IQR range)	10, 18.5 (4–22,5)	8, 20 (2–22)	

Regarding gastrointestinal complications, bowel complications requiring surgical revision (rBCs) were further analyzed as main end point in detail.

As a population of special interest according analysis were performed in the octogenarians (UCN 20 patients, IC 53 patients). The same patient's characteristics as in the general study population were extracted and can be seen in Table 2.

**Table 2:** Baseline characteristics in octogenarians.

**Urinary diversion type**	**UCN**	**IC**	***p*-value**
**Baseline**			
*n*	20	53	
Age, yr, median (range)	82.5 (80–89)	82 (80–89)	0.04
Body mass index, kg/m^2^, median (range)	25.5 (19–33)	25 (19.4–32)	0.72
Charlson-Comorbidity-Index, median (range)	6.5 (6–16)	6 (0–11)	0.89
Palliative intention, *n* (%)	9 (45)	5 (9.4)	0.00058
**Perioperative**			
Operation time, min, median (range)	216.5 (135–435)	275 (140–600)	0.038
Transfusion rate, *n* (%)	8 (40)	19 (36)	0.74
Blood loss, ml, median (range)	300 (0–800)	350 (0–4000)	0.63
Hospital stay, *d*, median	21 (6–64)	18 (10–100)	0.7
Intensive care unit stay, *n* (%)	4 (20)	12 (23)	0.81
Intensive care unit stay, d, median (range)	6 (1–9)	3 (1–24)	0.94
Pelvic lymph node dissection, *n* (%)	6 (30)	42 (79)	0.00008
Lymph node yield, *n*, median (range)	11 (2–19)	19 (1–47)	0.61
**Postoperative**			
Tumor stage, *n* (%)			
T0, ≤ T1	3 (15)	11 (21)	0.58
T2	4 (20)	9 (17)	0.76
T3	7 (35)	19 (36)	0.95
T4	6 (30)	14 (26)	0.76
Neoadjuvant chemotherapy, *n* (%)	0 (0)	3 (5.6)	

The retrospective data analysis of the Düsseldorf data set was approved by the institutional review board of the Düsseldorf University Hospital (study number 2020-1275). De-identified patient data from Cologne University was provided and processed in accordance with the Act on the Protection of Personal Data in the Health Care System (Gesundheitsdatenschutzgesetz—GDSG NW), §6 (data processing for scientific purposes).

### Statistical Analysis

Primary end point was the difference between rBCs in patients with IC vs. patients with UCN.

Descriptive statistics include median, range, and interquartile range (IQR). Statistical analysis was performed with Fisher exact test for categorical data (postoperative ICU stay, complications in Clavion–Dindo classification, rBC, transfusion rate, tumor stage) while continuous data (age, length of hospital stay, length of ICU stay, CCI, BMI, operation time, blood loss) were compared using two-tailed Mann–Whitney U test. The values of *p* < 0.05 were considered as statistically significant. Statistical tests were chosen after consultation with a statistician. All statistical tests were performed using GraphPad Prism^®^ v8.4.3.1 (GraphPad Software, La Jolla, CA, USA) and SPSS^®^ Statistics v27 (IBM Armonk, NY, USA). All tests were two-sided with a significance level set at *p* < 0.05.

## Results

### Patient's Characteristics

Between 2008 and 2020, 163 patients underwent radical cystectomy and either received an IC (*N* = 125) or a UCN (*N* = 38). Median follow-up was 20 months (2–22 months) in the UCN group and 18.5 months (4–22.5 months) in the IC group. Patients who received a UCN were older and presented with a higher CCI, though differences were not statistically significant (all *p* > 0.05) (see Table 1). Median age was 81 (range 75–89) years in the UCN group and 79 (range 75–89) years in the IC group. Median body mass index was 26 in both groups (UCN range 19–33, IC range 19–37). Median CCI was 7 (range 5–16) in the UCN group and 6 (range 0–11) in the IC group, respectively. Patients who received a UCN underwent surgery more frequently in palliative intention (UCN 37% vs. IC 10%, *p* < 0.05). There was no patient who received a neoadjuvant chemotherapy in the group of UCN while in the group of IC, there were 7 patients who received neoadjuvant chemotherapy.

### Perioperative Parameters and Postoperative Complication Rates

Regarding perioperative parameters, we observed a significantly shorter operation time in the UCN group [UCN 233 (range 130–450) min vs. IC 305 (range 100–600) min, *p* = 0.02]. Median blood loss [UCN 300 (0–1,200) ml vs. IC 300 (0–6,000) ml] and transfusion rate [UCN 12 (32%) vs. IC 33 (26%)] were comparable and not statistically significant. Median length of hospital stay also showed no difference [UCN 18 (IQR 14.75) days vs. IC 17 (IQR 10) days]. In case of the presence of urothelial carcinoma, tumor stage also showed no statistical difference with the majority of the patients presenting with T2-T3 urothelial carcinoma (UCN 71% vs. IC 67%). Pelvic lymph node dissection was performed in 16 patients in the UCN group (42%) and 99 patients in the IC group (79%, *p* < 0.05) with a median of 8 (UCN) and 17 (IC) removed lymph nodes, respectively (*p* < 0.05).

Regarding overall postoperative complications, the comparison of Clavien–Dindo classification showed no statistical difference between the two groups with an absolute count from Clavien–Dindo IIIA–IVB of 13 (34%) in the UCN group vs. 46 (37%) in the IC group (see Table 3). In the IC group, in total, 18 patients (14%) suffered from ureteric implantation stenosis requiring a consecutive operation or permanent ureteral stenting.

**Table 3:** Complications.

**Urinary diversion type**	**UCN**	**IC**
**Clavien-Dindo-Classification**, ***n*** **(%)**		
Clavien IIIA	8 (21)	14 (11)
Clavien IIIB	4 (11)	17 (14)
Clavien IVA	2 (5)	9 (7)
Clavien IVB	0 (0)	6 (5)
Clavien V	3 (8)	5 (4)

### Bowel Complications Requiring Surgical Revision

Nevertheless, rBC was a rare complication in UCN group (3/38, 8%) as compared to IC group (15/125, 12%, *p* > 0.05) (see Table 4). In the UCN group, 1 patient suffered a leakage of the rectum after intraoperative serosa lesion, 1 patient suffered rectum stump insufficiency after resection of the rectum for nonorgan-confined bladder cancer, and 1 patient suffered a volvulus due to candidemia. In the IC group, 5 patients suffered insufficiency of the ileal anastomosis, 1 patient experienced a stenosis of the ileal anastomosis followed by a mechanical ileus, 1 patient had a necrosis of the anastomosis followed by ileal resection, 5 patients suffered small intestine perforation, 2 patients developed a rectourethral fistula, and 1 patient suffered rectum stump insufficiency after resection of the rectum for invasive bladder cancer. Regarding the frequency of postoperative ICU stay, no differences were observed (UCN 16 vs. IC 16%). The 90-day mortality also did not differ in both groups (UCN 3/38, 8% vs. IC 5/125, 4%). In the UCN group, 2 patients died due to multiorgan failure due to sepsis and 1 patient died due to apoplex. In the IC group, 2 patients died due to myocardial infarction, 2 patients died due to apoplex, and 1 patient died because of multiorgan failure due to sepsis. One patient in the UCN group and two patients in the IC group died because of a bowel complication, one of the latter due to an insufficient ileal anastomosis.

**Table 4:** Bowel complications requiring revision.

**UCN**	
Patient 1	Volvulus due to candidemia
Patient 2	Leakage due to rectum deserosation
Patient 3	Rectum stump insufficiency after sigma resection due to cancer infiltration of the rectum
**IC**	
Patient 1	Small intestine perforation
Patient 2	Insufficiency of the ileal anastomosis
Patient 3	Small intestine perforation
Patient 4	Small intestine perforation
Patient 5	Small intestine perforation
Patient 6	Insufficiency of the ileal anastomosis
Patient 7	Small intestine perforation
Patient 8	Insufficiency of the ileal anastomosis
Patient 9	Insufficiency of the ileal anastomosis
Patient 10	Rectourethral fistula
Patient 11	Rectourethral fistula
Patient 12	Necrosis of the anastomosis followed by ileal resection
Patient 13	Stenosis of the ileal anastomosis followed by mechanical ileus
Patient 14	Rectum stump insufficiency
Patient 15	Insufficiency of the ileal anastomosis

### Analysis Results in Octogenarians

In a population of special interest, we evaluated complications in octogenarians (patients >80 years of age). Patients in the UCN group have been significantly older in this cohort (82.5 years vs. 82 years, *p* < 0.05). Operations in palliative intention were performed significantly more often in the UCN group (UCN 9/20, 45% vs. IC 5/53, 9%, *p* < 0.05). Congruent with this, pelvic lymph node dissection was performed less often in the UCN group (UCN 6/20, 30% vs. IC42/53, 79%, *p* < 0.05). Operation time was significantly shorter in the UCN group (216.5 vs. 275 min, *p* < 0.05).

In the UCN group, 1/20 patients (5%) had a bowel complication requiring revision while this was necessary in 5/53 patients (9%, *p* = 0.64) with IC. The patient in the UCN group suffered a volvulus due to candidemia. In the IC group, we monitored 3 insufficiencies of the ileal anastomosis, 1 small intestine perforation, and 1 rectourethral fistula (see Table 5).

**Table 5:** Bowel complications requiring revision in octogenarians.

**UCN**	
Patient 1	Volvulus due to candidemia
**IC**	
Patient 1	Small intestine perforation
Patient 2	Insufficiency of the ileal anastomosis
Patient 3	Insufficiency of the ileal anastomosis
Patient 4	Rectourethral fistula
Patient 5	Insufficiency of the ileal anastomosis

## Discussion

The aim of the study was to determine whether elderly patients benefit of receiving a UCN instead of an IC because of the higher risk the additional bowel procedure holds. The hypothesis was that this additional bowel procedure would increase life-threatening intestinal complications in this frail population as compared.

Physicians have to decide with the patient what kind of urinary diversion will be performed, yet there is no standard of care of which diversion is best in which patient. With a growing population of elderly patients, decision-making becomes more difficult. Although orthotopic neobladders should not be ruled out only because of age (2), incontinent urinary diversions is the procedure of choice (4). For the choice of the urinary diversion in our cohort, regular conferences were held to discuss the decision. Because of the lack of hard criteria for the choice of the urinary diversion, it has also to be discussed with the patient. Neurological or psychological illnesses, life expectancy, impaired liver or renal function, patient age, and intraoperative urothelial carcinoma-positive surgical margins are all reasons against more complex forms of diversion. Proper manipulation of the diversion has to be ensured. The EAU guideline also does not deliver a standard of choice for the urinary diversion (17).

In a retrospective analysis of 10,848 patients, Reese et al. (9) have shown that regardless of the urinary diversion, 633 patients (5.84%) needed an unplanned reoperation after radical cystectomy; 349 (60%) of those reoperations have been due to gastrointestinal complications. Fischer et al. (8) have shown 15% of anastomotic insufficiency after performing an additional intestinal procedure. In knowledge of this, when setting up this study, we were certain we would get a significant result concerning the numbers of bowel complications. Though in our retrospective analysis, we were not able to discern a statistical difference regarding the rate of severe intestinal complications in general. However, focusing on the individual patient level, rBCs in patients with UCN were preferably due to tumor burden requiring a surgical approach to the rectum while 7/15 patients in the IC group suffered from direct complications of the ileal anastomosis. Furthermore, one of the patients who received an anastomosis-related complication died. These absolute numbers indicate the risk of the additional bowel procedure.

Regarding the risk of bleeding, we could show that there is no higher risk of bleeding in the IC group, which supports the fact that bleeding is not a complication of the urinary diversion but of the radical cystectomy itself. Moreover, patients may have an additional advantage due to a significant shorter operation time when receiving a UCN, although a direct relation was not obvious in this study. In general, postoperative complications are comparable to current literature (18).

Although UCN has been performed more frequently for patients in a palliative setting as compared to the IC group, none of these patients died in direct context to the operation. This indicates that the risk of complications may be kept low by choosing a quicker and less burdensome operation technique. Yet it still has to be taken into account that also the tumor stadium plays a pivotal role in the complication rate of radical cystectomies. Two of the listed patients who suffered rectal injury in the UCN group presented a T4 tumor stage advancing into close proximity toward the rectum. The preoperative diagnostics therefore are of highest interest for the surgeon and the preparation of the operation. Maisch et al. (19) have shown that cystectomies in locally advanced pT4 bladder cancer is associated with an increased 90-day mortality rate; moreover, these findings go in line with the data of Isbarn et al. (20) who showed that not only age, but also tumor stage and histological subtype represent independent risk factors of perioperative mortality in bladder cancer. With this background, our data fit well in the existing context of current literature.

Due to the retrospective manner of the study, we are not able to present data of frailty scores as these have not been collected. In our study, we focused on elderly patients ≥75 years. Yet, age alone is not the factor that determines the outcome of such operations. Hanna et al. (21) have presented that targeted interventions such as prehabilitation in frail patients can lead to a better outcome. Frailty rises with increasing age (22) and as the population grows older, we are more and more confronted with frail patients. Therefore, we suggest that in elderly patients, a frailty score should be used and an elaborate discussion about UCN in frail patients should held. Moreover, the CCI score may be established preoperatively to improve the patient's selection in order to estimate the probability of postoperative complications (18).

Data about ureteral stenoses after IC have been collected; 18/125 (14%) of the IC patients presented with ureteral implantation stricture or required permanent ureter stenting after unsuccessful stent removal. This is slightly higher than the reported data in current literature which range from 4.3% to 9.1% (7, 23), yet it is in range for ureteroenteric strictures after urinary diversion in common [up to 15% (24)]. The often-mentioned advantage that the IC does not require permanent stenting is therefore not fulfilled for all patients, which may support the decision for a UCN in elderly patients. Though surgical correction may be successfully in more than half of patients, especially in the elderly population this may not be a valid option (25).

The population of special interest also showed no difference in the frequency of occurrence of bowel complications. This is in line with the publication of Haden et al. who showed that comparing septuagenarians to octogenarians in regard to their postoperative complications, there were no statistical differences. However, octogenarians have a higher mortality compared to septuagenarians after radical cystectomy (26). In our population, it is striking that UCN has been chosen in patients with palliative intention where a pelvic lymph node dissection was not performed. It is no surprise that such an operation leads to a significantly shorter operation time. The reversal is that those patients suffer less complications, as longer operation times lead to higher rates of complications (27).

## Limitations

Our study faces some limitations. The retrospective study setting is to mention first. Due to this, we cannot deliver data like quality of life or frailty as data required to perform accordant evaluations could not be retrieved in every case. Further, while postoperative complications have been thoroughly documented and are available for analyses, structured documentation of intraoperative complications has not been performed for all patients, especially in the first years we started our databases. Accordingly, a valid analysis in this context is not possible. A surgeon's bias can moreover not be ruled out as the surgeons (as well as experience and background such as number of surgeons) have not been taken into account. Possible confounders cannot be identified due to the statistical means. Moreover, patient selection was not standardized following a formal process but discussed in case-based conferences with surgical experts in this area. Selection of urinary diversion therefore was no objective decision. The limited sample number as well as the restriction to a bicentric study narrows statistical analysis and restricts both data interpretability and generalizability.

## Conclusion

Especially in elderly patients and in the palliative setting, performing a UCN is reasonable choice as a number of specific complications related to ICs may be avoided. However, clear indicators for patient selection have not yet been defined. In elderly patients, not only age as itself, but also frailty scores should be considered as an important preoperative parameter.

## Data Availability Statement

The original contributions presented in the study are included in the article/supplementary material, further inquiries can be directed to the corresponding author.

## Ethics Statement

The retrospective data analysis of the Düsseldorf data set was approved by the institutional review board of the Düsseldorf University Hospital (study number 2020-1275). De-identified patient data from Cologne University was provided and processed in accordance with the Act on the Protection of Personal Data in the Health Care System (Gesundheitsdatenschutzgesetz - GDSG NW), §6 (Data processing for scientific purposes). Written informed consent for participation was not required for this study in accordance with the national legislation and the institutional requirements.

## Author Contributions

All authors listed have made a substantial, direct, and intellectual contribution to the work and approved it for publication.

## Conflict of Interest

The authors declare that the research was conducted in the absence of any commercial or financial relationships that could be construed as a potential conflict of interest.

## Publisher's Note

All claims expressed in this article are solely those of the authors and do not necessarily represent those of their affiliated organizations, or those of the publisher, the editors and the reviewers. Any product that may be evaluated in this article, or claim that may be made by its manufacturer, is not guaranteed or endorsed by the publisher.

## References

[1] Richters A Aben KKH Kiemeney L. The global burden of urinary bladder cancer: an update. World J Urol. (2020) 38:1895–904. 10.1007/s00345-019-02984-4 31676912 PMC7363726

[2] Thurairaja R Burkhard FC Studer UE. The orthotopic neobladder. BJU Int. (2008) 102:1307–13. 10.1111/j.1464-410X.2008.07975.x 19035897

[3] Seer-Database (2021). Cancer of the Urinary Bladder-SEER Stat Fact Sheets. Available online at: https://seer.cancer.gov/statfacts/html/urinb.html (accessed 6th May 2021).

[4] Siddiqui KM Izawa JI. Ileal conduit: standard urinary diversion for elderly patients undergoing radical cystectomy. World J Urol. (2016) 34:19–24. 10.1007/s00345-015-1706-1 26475274

[5] Shabsigh A Korets R Vora KC Brooks CM Cronin AM Savage C. Defining early morbidity of radical cystectomy for patients with bladder cancer using a standardized reporting methodology. Eur Urol. (2009) 55:164–74. 10.1016/j.eururo.2008.07.031 18675501

[6] Patel HD Ball MW Cohen JE Kates M Pierorazio PM Allaf ME. Morbidity of urologic surgical procedures: an analysis of rates, risk factors, and outcomes. Urology. (2015) 85:552–9. 10.1016/j.urology.2014.11.034 25733265 PMC4349385

[7] Prcic A Begic E. Complications after ileal urinary derivations. Med Arch. (2017) 71:320–4. 10.5455/medarh.2017.71.320-324 29284898 PMC5723179

[8] Fischer N Ellinger J Koeditz B Heidenreich A Hoffmann MA. Predictors for outcome and complications related to urinary diversion. Anticancer Res. (2021) 41:5585–91. 10.21873/anticanres.15372 34732429

[9] Reese SW Ji E Paciotti M Leow JJ Mahvi DA Steele G. Risk factors and reasons for reoperation after radical cystectomy. Urol Oncol. (2020) 38:269–77. 10.1016/j.urolonc.2019.10.011 31761610

[10] Cicione A De Nunzio C Lombardo R Trucchi A Manno S Lima E. Complications and quality of life of ileal conduit, orthotopic neobladder and ureterocutaneostomy: systematic review of reports using the Clavien-Dindo Classification. Minerva Urol Nefrol. (2020) 72:408–19. 10.23736/S0393-2249.20.03641-3 32734749

[11] Erlich A Zlotta AR. Treatment of bladder cancer in the elderly. Investig Clin Urol. (2016) 57 Suppl 1:S26–35. 10.4111/icu.2016.57.S1.S26 27326404 PMC4910758

[12] Wang GJ Barocas DA Raman JD Scherr DS. Robotic vs open radical cystectomy: prospective comparison of perioperative outcomes and pathological measures of early oncological efficacy. BJU Int. (2008) 101:89–93. 10.1111/j.1464-410X.2007.07212.x 17888044

[13] Zebic N Weinknecht S Kroepfl D. Radical cystectomy in patients aged > or = 75 years: an updated review of patients treated with curative and palliative intent. BJU Int. (2005) 95:1211-4. 10.1111/j.1464-410X.2005.05507.x 15892803

[14] Bream MJ Maurice MJ Altschuler J Zhu H Abouassaly R. Increased use of cystectomy in patients 75 and older: a contemporary analysis of survival and perioperative outcomes from the national cancer database. Urology. (2017) 100:72–8. 10.1016/j.urology.2016.08.054 27765588

[15] Atallah F Letocart P Malavaud B Ahmad M Mazerolles M Minville V. Can we predict morbidity and mortality of patients aged 75 years and older undergoing cystectomy? J Frailty Aging. (2017) 6:72–5. 10.14283/jfa.2017.5 28555706

[16] Richards KA Kader AK Otto R Pettus JA Smith JJ 3rd Hemal AK. (2012). Is robot-assisted radical cystectomy justified in the elderly? A comparison of robotic versus open radical cystectomy for bladder cancer in elderly >/=75 years old. J Endourol. 26:1301–06. 10.1089/end.2012.0035 22582706

[17] Witjes JA Bruins HM Cathomas R Comperat EM Cowan NC Gakis G. European association of urology guidelines on muscle-invasive and metastatic bladder cancer: summary of the 2020 guidelines. Eur Urol. (2021) 79:82–104. 10.1016/j.eururo.2020.03.055 32360052

[18] Vetterlein MW Klemm J Gild P Bradtke M Soave A Dahlem R. Improving estimates of perioperative morbidity after radical cystectomy using the european association of urology quality criteria for standardized reporting and introducing the comprehensive complication index. Eur Urol. (2020) 77:55–65. 10.1016/j.eururo.2019.08.011 31473012

[19] Maisch P Lunger L Duwel C Schmid SC Horn T Gschwend JE. Outcomes of palliative cystectomy in patients with locally advanced pT4 bladder cancer. Urol Oncol. (2021) 39:368 e311–7. 10.1016/j.urolonc.2020.11.042 33431328

[20] Isbarn H Jeldres C Zini L Perrotte P Baillargeon-Gagne S Capitanio U. A population based assessment of perioperative mortality after cystectomy for bladder cancer. J Urol. (2009) 182:70–7. 10.1016/j.juro.2009.02.120 19447427

[21] Hanna K Ditillo M Joseph B. The role of frailty and prehabilitation in surgery. Curr Opin Crit Care. (2019) 25:717–22. 10.1097/MCC.0000000000000669 31689246

[22] Dent E Martin FC Bergman H Woo J Romero-Ortuno R Walston JD. Management of frailty: opportunities, challenges, and future directions. Lancet. (2019) 394:1376–86. 10.1016/S0140-6736(19)31785-4 31609229

[23] Roghmann F Gockel M Schmidt J Hanske J Von Landenberg N Loppenberg B. Complications after ileal conduit: Urinary diversion-associated complications after radical cystectomy. Urologe A. (2015) 54:533–41. 10.1007/s00120-015-3812-5 25895565

[24] Goh AC Belarmino A Patel NA Sun T Sedrakyan A Bochner BH. A population-based study of ureteroenteric strictures after open and robot-assisted radical cystectomy. Urology. (2020) 135:57–65. 10.1016/j.urology.2019.07.054 31618656

[25] Van Son MJ Lock M Peters M Van De Putte EEF Meijer RP. Treating benign ureteroenteric strictures: 27-year experience comparing endourological techniques with open surgical approach. World J Urol. (2019) 37:1217–23. 10.1007/s00345-018-2475-4 30232554 PMC6533231

[26] Haden TD Prunty MC Jones AB Deroche CB Murray KS Pokala N. Comparative perioperative outcomes in septuagenarians and octogenarians undergoing radical cystectomy for bladder cancer-do outcomes differ? Eur Urol Focus. (2018) 4:895–9. 10.1016/j.euf.2017.08.005 28865996

[27] Cheng H Clymer JW Po-Han Chen B Sadeghirad B Ferko NC Cameron CG. Prolonged operative duration is associated with complications: a systematic review and meta-analysis. J Surg Res. (2018) 229:134–44. 10.1016/j.jss.2018.03.022 29936980

